# Incidence, risk factors and ophthalmic clinical characteristic of ethambutol-induced optic neuropathy: 7-year experience

**DOI:** 10.3389/fopht.2023.1152215

**Published:** 2023-03-10

**Authors:** Pareena Chaitanuwong, Supaporn Srithawatpong, Paisan Ruamviboonsuk, Supanut Apinyawasisuk, Akechanok Watcharapanjamart, Heather E. Moss

**Affiliations:** ^1^ Ophthalmology Department, Rajavithi Hospital, Ministry of Public Health, Bangkok, Thailand; ^2^ Department of ophthalmology, Faculty of Medicine, Rangsit University, Bangkok, Thailand; ^3^ Ophthalmology Department, King Chulalongkorn Memorial Hospital, Thai Red Cross Society, Bangkok, Thailand; ^4^ Department of Ophthalmology, Faculty of Medicine, Chulalongkorn University, Bangkok, Thailand; ^5^ Department of Ophthalmology, Stanford University, Palo Alto, California, CA, United States; ^6^ Department of Neurology & Neurological Sciences, Stanford University, Palo Alto, California, CA, United States

**Keywords:** ethambutol, optic neuropathy, drug toxicity, visual impairment, tuberculosis

## Abstract

**Background:**

The purpose of this research was to investigate the characteristics, clinical manifestations, incidence, and risk factors in ethambutol-induced optic neuropathy (EON) in the Thai population.

**Methods:**

Patients treated with ethambutol for tuberculosis (TB) were retrospectively identified in the medical record of a tertiary hospital in Thailand from January 2012 to August 2019. Development of EON was determined through review of ophthalmology records. Comparison was made between patients with EON and those without EON to identify possible risk factors. Ophthalmic outcomes were characterized.

**Results:**

Among 4,141 patients who received ethambutol for TB treatment, 1,062 had an ophthalmology encounter, and 20 (0.5% overall, 1.88% with ophthalmology encounters) developed EON. In unadjusted analysis, compared to patients without EON, those with EON had a similar daily dose, but longer duration of ethambutol treatment (P=0.02). They were older (mean 43.74 vs. 58.60 years, P=0.001), more likely to have hypertension (P=0.02) and smoke (p=0.01). There were no differences in gender, body mass index, diabetes, dyslipidemia, HIV infection or glomerular filtration rate. The peripapillary retinal nerve fiber layer, ganglion cell analysis, and vascular density as measured using retinal optical coherence tomography were impacted by EON. In adjusted logistic regression analysis, age greater than 60 (OR = 8.71, p = 0.01) and smoking (OR = 7.06, p = 0.01) were independent risk factors for EON.

**Conclusion:**

In patients treated with ethambutol, the incidence proportion of EON was 0.5% among those with ethambutol administered and 1.88% among those with ethambutol and an eye visit. Potential EON risk factors were age, hypertension, smoking, and duration of ethambutol medication. Smoking has not been associated with EON in prior studies.

## 1 Introduction

Tuberculosis (TB) has long been a global public health problem with both health and socioeconomic consequences. Thailand is one of the 30 nations with highest prevalence of TB and HIV/TB co-infection (1). Anti-TB drugs are associated with increased survival of tuberculosis patients. However, side effects, including toxicity to the visual system, impact quality of life and can lead to chronic disability.

Ethambutol is a bacteriostatic antibiotic that is used to treat Mycobacterium strains in combination with other medications. Ethambutol-induced optic neuropathy (EON) is one of the most common and devastating side effects (2). Patients with EON present with subacute painless bilateral visual deterioration, typically starting 2-8 months after initiation of ethambutol administration. Common examination findings are bilateral visual acuity decline, central or cecocentral visual field defect, normal optic disc appearance or mild hyperemic swelling in an early stage, evolving to pallor in a chronic stage. The condition may result in permanent visual impairment if cessation of the causative medication is delayed.

There have been several studies on the occurrence and the clinical presentation of EON, but none of them were undertaken in Southeast Asia, where tuberculosis is common (3–7). The purpose of this study was to investigate the incidence, clinical characteristics, and possible risk factors for developing EON in a tertiary care center in Southeast Asia.

## 2 Materials and methods

This study is a retrospective observational study of patients who received ethambutol for TB treatment seen at Rajavithi Hospital, Bangkok, Thailand from January 2012 to August 2019. The study protocol was approved by the Institutional Ethics Committee of Rajavithi hospital. The study was registered to the Thai Clinical Trials Registry with a registration number of TCTR20220207006.

### 2.1 Subjects

Patients who received ethambutol for TB standardized treatment regimen which is usually not exceed 25 mg/kg/d at Rajavithi Hospital, Bangkok, Thailand from January 2012 to August 2019 were identified from the hospital database. Subjects were grouped as ethambutol-induced optic neuropathy (EON) and non-EON. Inclusion criteria for EON were ICD-10 code H47 (other disorders of optic nerve and visual pathways) in the hospital database and confirmed as incident EON during the study period by chart review. The patients who received ethambutol during the study period without confirmed EON were classified as control (non-EON). Non-EON patients with an ophthalmology encounter during the study period were classified as controls with eye examination (non-EON with eye encounter). 173 non-EON patients with eye encounters were selected at random for review of ophthalmology records in order to validate the classification of non-EON status. Exclusion criteria for all subjects were incomplete or lost medical record.

### 2.2 Variable definition

For all subjects, demographic data including age at the time ethambutol treatment was started and gender were collected. Primary target organ of TB infection, total duration and daily dose of ethambutol were extracted from the hospital data base. Body mass index (BMI) at time of starting drug, and history of cigarette smoking were also extracted. Medical history including the presence of other underlying medical conditions such as hypertension, diabetes mellitus, dyslipidemia and HIV infection were noted.

In EON subjects, ophthalmic data, including visual acuity, pattern of visual field defect, color vision, and optic disc abnormalities were extracted from the medical record. The results of optical coherence tomography (OCT), optical coherence tomography angiography (OCTA), using SPECTRALIS^®^ (Heidelberg Engineering, Germany) were recorded when available.

### 2.3 Statistical analysis

EON incidence proportion (cumulative incidence) was calculated as the proportion newly diagnosed cases of EON during the defined period relative to total number of individuals treated with ethambutol for TB (EON + non-EON) and relative to the total number of individuals treated with ethambutol for TB who had an eye exam during the study period (EON + non-EON with eye encounter). To determine which factors were associated with EON, Chi-square test and Fishers’ Exact test were used for categorical variables, t-test for independent samples was used for the comparison of continuous variables. Logistic regression analysis was used to investigate all factors that differed between groups and may be linked to the development of EON. A p-value less than 0.05 was considered statistically significant. The data were analyzed using SPSS version 20.0 (IBM Inc.).

## 3 Results

### 3.1 Incidence of ethambutol-induced optic neuropathy (EON)

**Figure 1:**
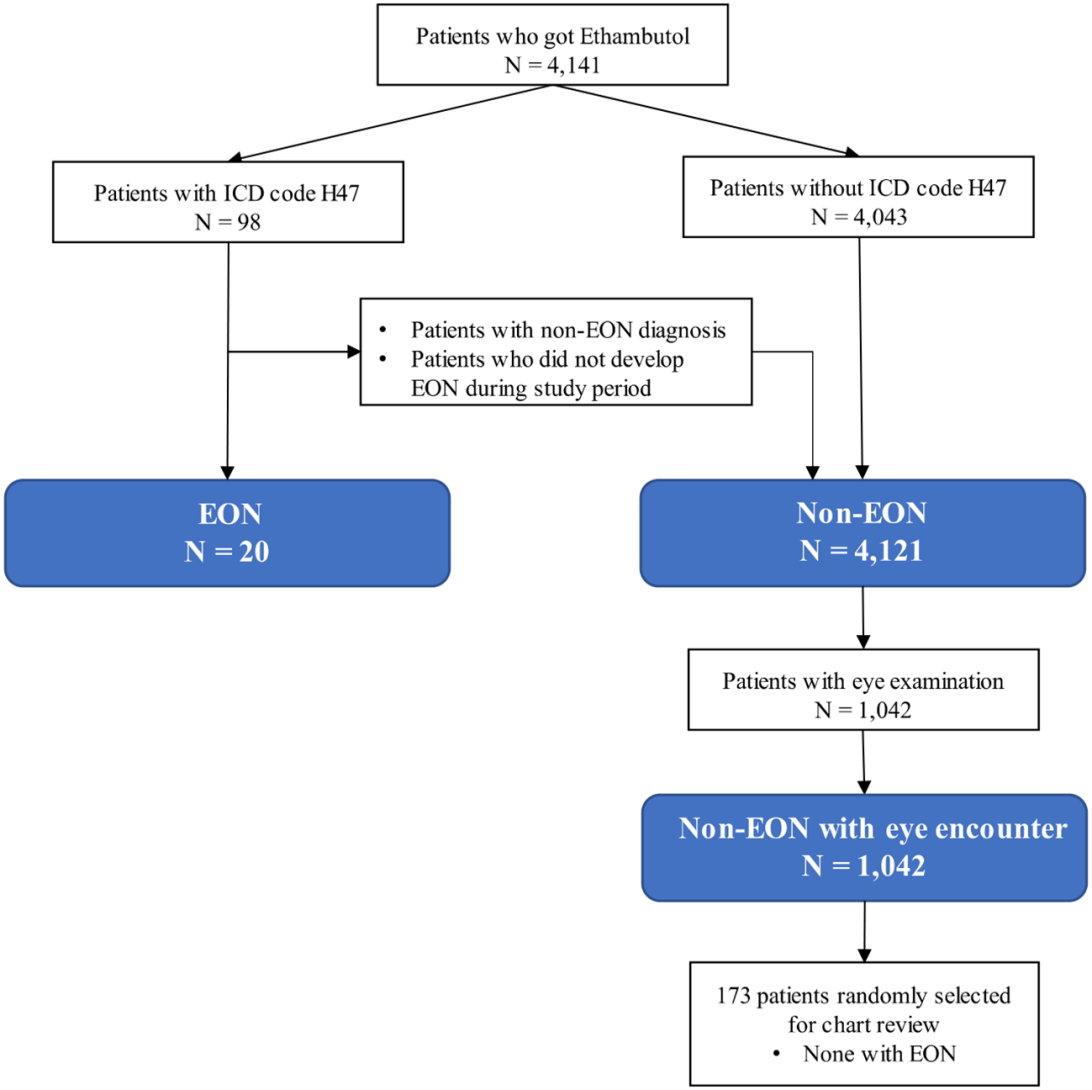
Flow diagram of patient selection.

Twenty (0.5%) of the 4,141 patients who received ethambutol for TB treatment during the study period were diagnosed as EON. Among the 1,062 patients with eye examinations incidence of EON was 1.88%. We randomly examined the medical records of 173 patients receiving ethambutol with eye exam and lacking ICD code H47 and confirmed that none of them were diagnosed with EON (**Figure 1**). Of the 20 EON patients, twelve patients had primary pulmonary tuberculosis, four had tuberculosis of the spine, one had tuberculosis of the knee, one had pleural tuberculosis, one had liver abscess tuberculosis, and one had tuberculosis meningitis (**Table 1**).

**Table 1:** Characteristics of ethambutol treated patients with and without EON.

	Non-EON (Controls) (n = 4121)	Non-EON (Controls) with eye examination (n = 1042)	EON cases (n = 20)
Gender			
**Male, n (%)**	2383 (57.8)	571 (55.9)	13 (65)
**Female, n (%)**	1738 (42.2)	450 (44.1)	7 (35)
**Age (years) (mean ± SD)**	43.74 ± 16.94	46.37 ± 16.31	58.60 ± 12.01^◊^
**Weight (Kg) (mean ± SD)**	61.47 ± 16.85	65.17 ± 18.77	57.50 ± 9.05
**BMI (kg/m^2^) (mean ± SD)**	22.92 ± 5.99	24.07 ± 6.69	20.72 ± 2.82
**Smoking, n (%)**	204 (4.9)	39 (7.4)	4 (20)^†^
**DM, n (%)**	458 (11.1)	274 (26.8)	5 (25)
**HTN, n (%)**	576 (13.9)	293 (28.7)	7 (35)*
**DLP, n (%)**	504 (12.2)	302 (29.6)	4 (20)
**HIV, n (%)**	628 (15.2)	309 (30.2)	2 (10)
**GFR (mL/min) (mean ± SD)**	99.21 ± 28.13	93.84± 29.49	87.81 ± 29.50
**EMB Dose(mg/kg/day) (mean ± SD)**	15.25 ± 3.54	14.49± 3.65	19.12 ± 3.89
**Duration of EMB prescription (days) (mean ± SD)**	106.94 ± 225.81	159.96 ± 306.89	235.60 ± 122.86^◊^

* P-value < 0.05 versus each control group in chi-square test, † P-value < 0.05 versus each control group in fisher exact test, ◊ P-value < 0.05 versus each control group in t-test.

EON, ethambutol optic neuropathy; BMI, Body Mass Index; DM, Diabetes Mellitus; HTN, Hypertension; DLP, Dyslipidemia; HIV, Human immunodeficiency virus; GFR, Glomerular filtration rate; EMB, ethambutol.

The ages of EON affected patients ranged between 27 and 74 years (mean 58.6 ± 12.0 years); 13 were men and 7 were women. The average BMI was 20.7 ± 2.8 Kg/m^2^ (n=16). There were 5 (25%) patients with diabetes mellitus, 7(35%) patients with hypertension, 4(20%) patients with dyslipidemia. There was no HIV patient in the EON affected group. 4(20%) patients had a history of cigarette smoking. The average glomerular filtration rate (GFR) was 87.8 ± 29.5 mL/min. The mean dose and duration of ethambutol were 19.1 ± 3. 9 mg/kg/day and 235.6 ± 122.9 days, respectively (**Table 1**).

### 3.2 Unadjusted comparison

**Figure 2:**
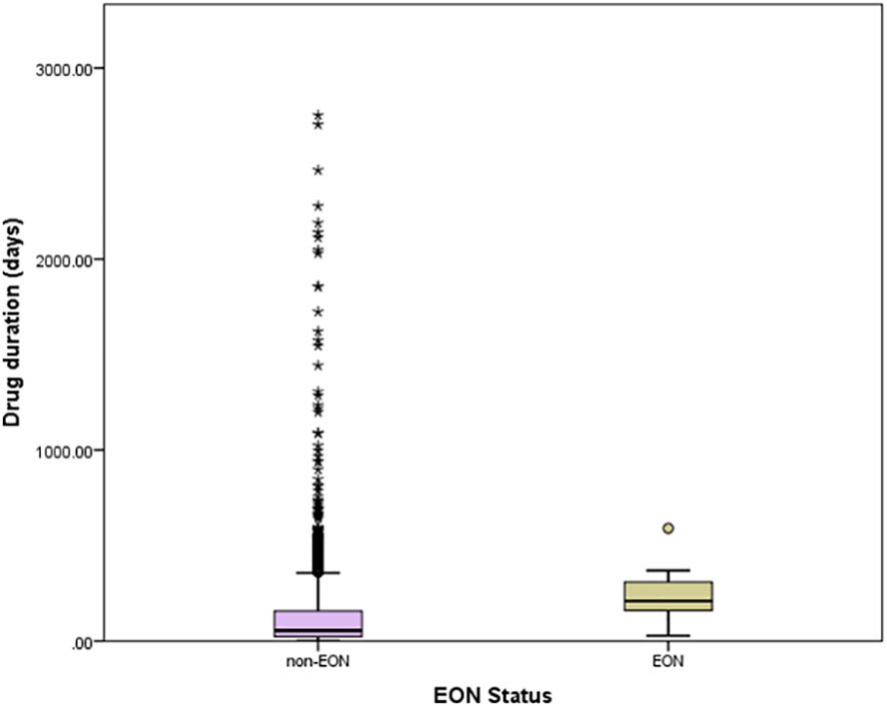
Distribution of ethambutol treatment duration according to EON status (all patients). Box boundaries are 25%ile and 75%ile, error bars are 5%ile and 95%ile, other symbols are cases between 95%ile and 100%ile.

In unadjusted analysis, patients with EON were older than those without EON, regardless of comparison group (P = 0.001, t-test). Body weight and BMI were not different between EON and non-EON groups. Smoking was more common in those with EON, regardless of comparison group (P=0.01, fisher exact). When comparing patients with EON to those without EON, hypertension was more common (P =0.02, chi-square). There was no difference between prevalence of diabetes, dyslipidemia, and HIV infection between those with and without EON. GFR did not differ between patients with and without EON (**Table 1**). There was no difference between the daily ethambutol doses delivered to patients with and without EON, with (P = 0.66, t-test) or without an eye exam (P =0.27, t-test). The duration of ethambutol therapy was longer (P =0.01, t-test) in patients with EON than those without EON (**Figure 2**).

### 3.3 Multiple variable models of EON

Age, smoking, hypertension and duration of ethambutol treatment were included in the logistic regression model due to their unadjusted associations with EON status. The incidence of EON was considerably higher in patients over the age of 60, as shown by logistic regression analysis (**Table 2**). With an odds ratio of 8.71 (P =0.01), patients over the age of 60 had a higher incidence of EON than those under the age of 40. Smoking status also remained independently associated in the adjusted model (OR 7.06, P=0.01). Hypertension was also associated with EON in the adjusted model but did not reach the statistical significance threshold (P=0.14). Duration of ethambutol was not associated with EON after adjusting for other variables (P=0.06).

**Table 2:** Logistic regression analysis of the factors for Ethambutol-induced Optic neuropathy.

	Unadjusted OR (95%CI) p-value	Adjusted OR (95%CI) p-value
**Age group** < 40 years	Ref	Ref
40-60 years	4.92 (1.02-23.71) 0.05	1.75 (0.28-10.84) 0.55
>60 years	14.23 (3.15-64.36) 0.001	8.71 (1.65-45.93) 0.01
**Smoking**	5.20 (1.55-17.43) 0.007	7.06 (2.00-24.87) 0.01
**Hypertension**	3.31 (1.32-8.34) 0.01	2.39 (0.65-8.80) 0.14
**Duration of Ethambutol**	1.01 (1.00-1.02) 0.03	1.00 (0.98-1.03) 0.06

### 3.4 Ophthalmic clinical characteristics

All of the EON patients had symptoms of bilateral painless visual loss. Visual acuity ranged from NPL to 20/20 at presentation. 2 patients (10%) had only a slight decrease in visual acuity but showed abnormalities in their visual fields.10 patients who were able to take a color test were found to have a color deficiency. The most common visual field defects were central or cecocentral scotomas [3 patients (15%)] and bitemporal scotomas [2 patients (10%)].

The duration of ophthalmic follow-up following EON diagnosis at eye appointment ranged from 2 to 83 months (21.7± 25.2). After stopping ethambutol, the visual function of 12 patients (60%) improved by at least 2 lines on the Snellen chart, with a mean recovery time of 6.9 months. There were no differences in demographics, past medical history, smoking, or ethambutol dosing between patients who did and did not recover. Follow up time in non-recovery patients was shorter than for patients with recovery (mean 7.7 months vs. 33.21, P=0.02).

**Figure 3:**
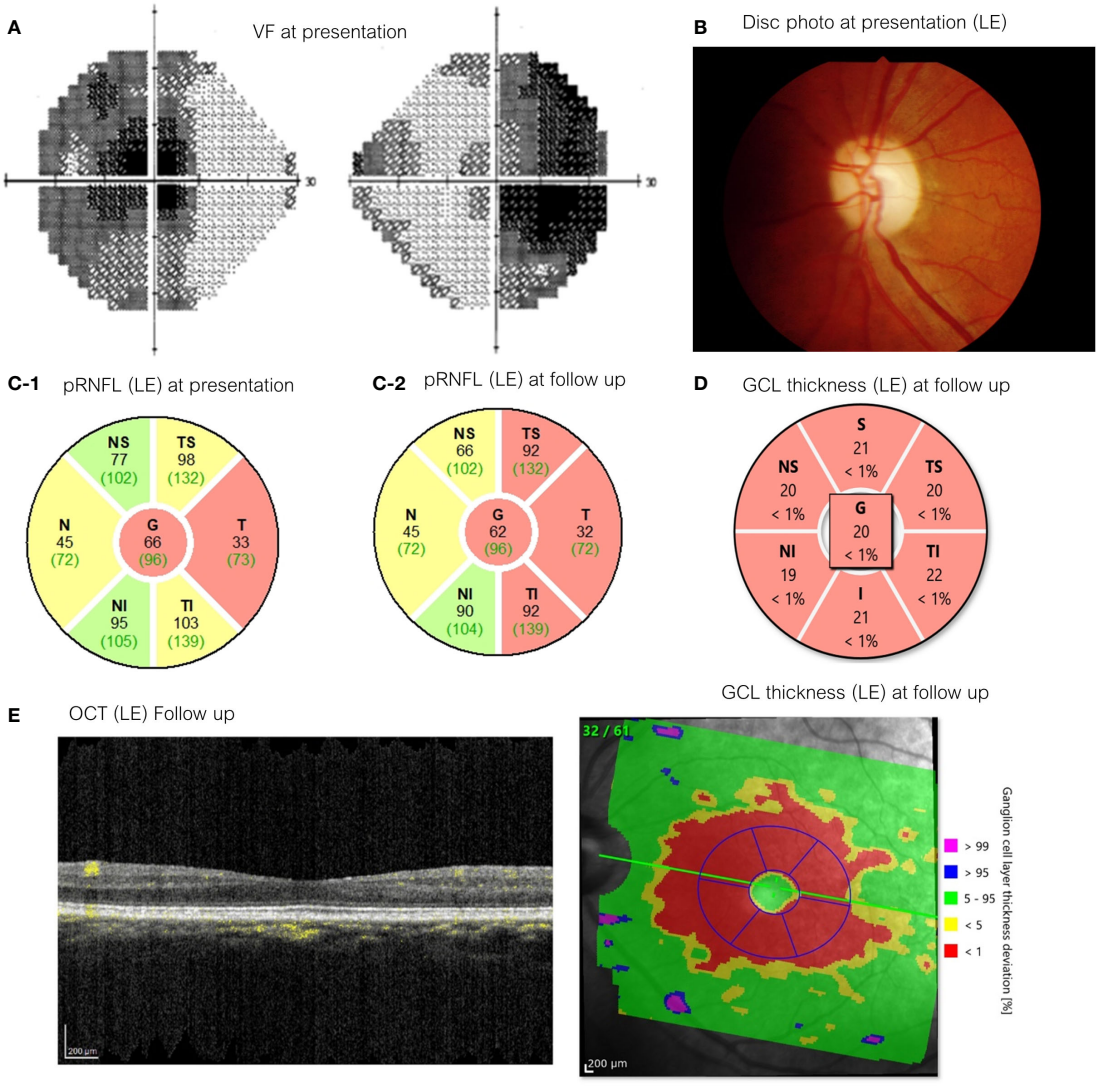
A 54-year-old woman with spine tuberculosis examined after 6 months of EMB medication (presentation) with best-corrected visual acuity 20/800 in the right eye and CF2’ in the left eye and 4 years after drug cessation (follow up) with VA 20/40 in both eyes. **(A)** Visual field testing showed the presence of Bitemporal hemianopia (presentation) **(B)** Optic disc appearances on her left eye showed mild temporal pallor (presentation). **(C)** pRNFL thickness left eye [C-1: presentation (first visit), C-2: follow up]. **(D)** GCL thickness of left eye (follow up). **(E)** OCT appearance of her left eye (follow up). EMB, ethambutol; CF, Counting fingers; pRNFL, peripapillary retinal nerve fiber layer; GCL, ganglion cell layer; OCT, Optical coherence tomography.

Ophthalmic imaging was available in 9 EON cases. These showed a decrease in the thickness of the peripapillary retinal nerve fiber layer (pRNFL) and macular ganglion cell layer (GCL), with the latter being more pronounced. In 2 chronic EON patients, characterized by pallor of the disc, OCTA revealed a loss of vessel density in macula whole area (6x6 mm) in both superficial and deep layers. Imaging follow-up was available in 3 cases and revealed minimal progression of pRNFL and GCL loss after ethambutol discontinuation. 2 cases with profound ganglion cell thinning at time of diagnosis, had visual function recovery after ethambutol discontinuation (**Figure 3**).

## Discussion

At a tertiary care medical center in Thailand the incidence of ethambutol optic neuropathy from 2012-2019 was 0.5% among all patients who received ethambutol for TB and 1.88% among patients who received ethambutol for TB with eye examination. In comparison, a recent Taiwanese study reported a 1.29% incidence of EON (8), while earlier studies reported an incidence of around 1% (6) (**Table 3**). Our overall incidence may be an underestimate due to missing hospital records, undetected diagnoses from other hospitals, or undiagnosed disease.

**Table 3:** Table comparing the literature on ethambutol optic neuropathy.

First Author (year)	Country	Study design	Participants	Study time (y)	Incidence Proportion % (cumulative incidence), (incident cases/cohort size)	Factors associated with EON	Visual outcomes
**Yang (2016) (** 3 **)**	Korea	Retrospective	480 non-HIV TB on EMB-containing multidrug regimens	6	0.70% (3/480)	- Age	3 cases presented with EON. Retinal nerve fiber layer thinning in 2 cases
**Chen (2012) (** 4 **)**	Taiwan	Retrospective	11,753 subjects who were dispensed EMB	8	2% (231/11,753)	- Age - Hypertension - Renal diseases	not reported
**Lee (2008) (** 5 **)**	Korea	Retrospective	857 patients who started ethambutol treatment for TB infection	2	1.50% (13/857)	- Renal dysfunction - Daily dose of ethambutol	Visual function after discontinuation of ethambutol is reversible in only a minority of patients.
**Talbert (2010) (** 6 **)**	USA	Case series and Meta-analysis	70 cases EON (16 + 54 review)	8	not reported	- Age - Renal dysfunction - Ethambutol dose - Ethambutol duration	not reported
**Ezer (2013) (** 7 **)**	Canada	Systematic review	19 studies before 2011	46	2.02% (102/5042)	No relevant significant factors were found.	not reported
**Chen (2015) (** 8 **)**	Taiwan	Retrospective	4803 newly diagnosed tuberculosis cases	10	1.29% (62/4803)	not reported	No factors associated with visual recovery.

We found older age to be associated with EON, which is consistent with prior reports (5, 6, 9–11). A recent study by Chen HY, et al. in a Taiwanese population reported that the majority of EON patients are over the age of 65, which is consistent with our findings (4). They hypothesized that age was an independent risk factor for EON mediated by renal function decline (6, 12). Although it is known that renal tubular function declines with age, the aging effect of ethambutol clearance is unknown (12). We also found a lower GFR in the EON group, but it did not reach statistical significance.

Hypertension and smoking were associated with EON in our study. Chen HY et al. study also found that hypertension was associated with EON and hypothesized that the effect was mediated through reduced renal function (4). Smoking is a potential risk factor that has not been reported in association with EON (**Table 3**). However, there is literature supporting smoking as a risk factor for TB infection, increasing the risk of recurrent TB, and impairing response to disease treatment (13). As a result, we hypothesize that smoking could increase the risk of EON mediated by more severe TB infection perhaps impacting drug regimen in patients who smoke. Another possibility is the adverse effect of smoking on mitochondrial function, which has been proposed as the mechanism for worsening Leber’s hereditary optic neuropathy (14). This mechanism could both increase the risk of developing EON and worsen the prognosis of EON.

According to a previous meta-analysis (6), duration and dose of ethambutol were positively related to the risk of EON. In our study, the ethambutol duration was longer in those with EON, though this association did not persist in adjusted models. While the dose in the EON group was higher than in the non-EON group, the difference did not reach statistical significance. This may be because the prescribed ethambutol regimen (15-25 mg/kg/d) was standardized to reduce the risk of EON (15, 16). We hypothesized that because of this standard dosing regimen, duration, rather than higher daily doses of ethambutol, drive total exposure.

The ophthalmic EON characteristics in our study were similar to those in previous studies. Patients with EON typically present with symmetrical bilateral painless visual loss. The most common visual field defects were central or cecocentral scotoma and bitemporal scotoma. We discovered that in 60% of cases, visual function recovered. Chamberlain PD, et al. study’s found that patients’ recovery rates ranged from 20% to 80%, which is consistent with our findings (3).

There are some limitations that should be mentioned. First, the data of the patients’ diagnosis as EON was initially identified using ophthalmology clinicians *via* ICD-10 coding, which could miss cases due to coding errors or not having an eye exam. Second, because this was a retrospective study, selection bias is likely. Third, our sample size is limited, because the computer-based diagnosis was only available at our institution for the 7 years studied. Fourth, because some clinical data, such as special ophthalmic imaging values, were not collected from all EON patients, we were unable to perform a comparative analysis. Fifth, Thailand’s standard treatment for tuberculosis during the study perior was multi-drug therapy. In this study, ethambutol optic neuropathy was diagnosed clinically, imaging was used to rule out other causes, and the patients’ vision did not worsen after ethambutol was discontinued. However, we cannot rule out other potential causes of optic neuropathy.

In conclusion, the incidence proportion (cumulative incidence) of EON in patients treated with Ethambutol was 0.5%-1.88% depending on the comparison group used. Age, hypertension, smoking, and the duration of ethambutol treatment were associated with EON in unadjusted analysis. Logistic regression showed age greater than 60 and smoking to persist as independent associations after adjustment for other covariates. Further study is needed to confirm smoking as a risk factor and consideration should be given to addressing this potentially treatable risk factor.

## Data availability statement

The original contributions presented in the study are included in the article/Supplementary Material. Further inquiries can be directed to the corresponding author.

## Author contributions

PC, SS, and PR contributed to conception and design of the study. AW organized the database. PC and AW performed the statistical analysis. PC and AW wrote the first draft of the manuscript. PC, PR, SA, and HM wrote sections of the manuscript. HM revised the manuscript as the most recent submission version. All authors contributed to manuscript revision, read, and approved the submitted version.
